# Identification of High-Risk Plaques by MRI and Fluorescence Imaging in a Rabbit Model of Atherothrombosis

**DOI:** 10.1371/journal.pone.0139833

**Published:** 2015-10-08

**Authors:** Ning Hua, Fred Baik, Tuan Pham, Alkystis Phinikaridou, Nick Giordano, Beth Friedman, Michael Whitney, Quyen T. Nguyen, Roger Y. Tsien, James A. Hamilton

**Affiliations:** 1 Department of Physiology and Biophysics, Boston University School of Medicine, Boston, Massachusetts, United States of America; 2 Division of Head and Neck Surgery, University of California at San Diego, La Jolla, California, United States of America; 3 Department of Biomedical Engineering, Boston University, Boston, Massachusetts, United States of America; 4 Division of Imaging Sciences and Biomedical Engineering, King’s College London, London, United Kingdom; 5 Department of Pharmacology, University of California at San Diego, La Jolla, California, United States of America; 6 Howard Hughes Medical Institute, University of California at San Diego, La Jolla, CA, United States of America; Baker IDI Heart and Diabetes Institute, AUSTRALIA

## Abstract

**Introduction:**

The detection of atherosclerotic plaques at risk for disruption will be greatly enhanced by molecular probes that target vessel wall biomarkers. Here, we test if fluorescently-labeled Activatable Cell Penetrating Peptides (ACPPs) could differentiate stable plaques from vulnerable plaques that disrupt, forming a luminal thrombus. Additionally, we test the efficacy of a combined ACPP and MRI technique for identifying plaques at high risk of rupture.

**Methods and Results:**

In an atherothrombotic rabbit model, disrupted plaques were identified with *in vivo* MRI and co-registered in the same rabbit aorta with the *in vivo* uptake of ACPPs, cleaved by matrix metalloproteinases (MMPs) or thrombin. ACPP uptake, mapped *ex vivo* in whole aortas, was higher in disrupted compared to non-disrupted plaques. Specifically, disrupted plaques demonstrated a 4.5~5.0 fold increase in fluorescence enhancement, while non-disrupted plaques showed only a 2.2~2.5 fold signal increase. Receiver operating characteristic (ROC) analysis indicates that both ACPPs (MMP and thrombin) show high specificity (84.2% and 83.2%) and sensitivity (80.0% and 85.7%) in detecting disrupted plaques. The detection power of ACPPs was improved when combined with the MRI derived measure, outward remodeling ratio.

**Conclusions:**

Our targeted fluorescence ACPP probes distinguished disrupted plaques from stable plaques with high sensitivity and specificity. The combination of anatomic, MRI-derived predictors for disruption and ACPP uptake can further improve the power for identification of high-risk plaques and suggests future development of ACPPs with molecular MRI as a readout.

## Introduction

Cardiovascular disease (CVD) remains the leading cause of mortality and morbidity in developed countries [1]. Atherosclerosis is a major contributor for CVD, and its complex pathology encompasses many features, including retention of lipids, infiltration of macrophages, deposition and degradation of the extracellular matrix proteins (MMP), increased inflammation and enzymatic activity in the arterial wall [2,3]. Although atherosclerotic plaques develop a range of pathophysiological features, a simple functional classification would be to label a plaque as either “stable” or “high-risk/vulnerable”. Stable plaques can remain clinically silent for decades; however, vulnerable plaques may suddenly disrupt to form a luminal thrombus and lead to clinical manifestations, including myocardial infarction and stroke. The ability to determine whether or not a specific plaque is likely to disrupt will guide clinical treatment and decrease unnecessary, expensive, and sometimes highly invasive, treatment of stable plaques that would not cause a future cardiac event.

Recently, strategies employing targeted molecular probes in conjunction with various imaging techniques have been able to visualize specific biological processes in atherosclerosis, including extracellular matrix changes [4], macrophage infiltration [5], neovascularization [6] and enzymatic activity [7–9], all of which provide important information on plaque stability. Among these imaging modalities, MRI has been used, alone, to identify numerous plaque features, including plaque composition [10–12], vascular remodeling [13,14], endothelial shear stress [15] and neovascularization [16]. The potential of MRI for monitoring atherosclerosis could be enhanced by rationally designed molecular probes that are targeted to biomarkers known to be associated with plaque vulnerability.

Among the targets that were deemed to be highly active and physiologically relevant, MMPs and thrombin are of particular interest. MMPs and thrombin have been linked to both atherogenesis and plaque vulnerability [17]. MMPs are a family of proteases that degrade the extracellular matrix (ECM), which can destabilize the fibrous cap [18]. Higher MMP levels have been detected in regions of low endothelial shear stress, a characteristic of plaque progression and vulnerability, in animal models [15,19]. Increased MMP activity has also been detected in human plaques both *in vivo* and *ex* v*ivo* [20,21]. MMP levels have been shown to indirectly influence thrombin activity via platelet aggregation [18]. The enhanced thrombin activity is important in the initiation, progression, and destabilization of plaques [22]. Thrombin is a trypsin-like serine protease with an inactive precursor, prothrombin, and is well established as a key regulator of blood coagulation. Prothrombin is activated by factors released from epithelial cells and macrophages in atherosclerotic plaques [23]. This can cause plaque instability by inducing intraplaque hemorrhage or by increasing the local ECM degradation by activating MMPs [18,24,25]. Therefore, probes that target MMPs or thrombin may help better the understanding of atherosclerotic progression and provide a new tool for diagnosing and monitoring.

In this study, fluorescent-labeled Activatable Cell Penetrating Peptides (ACPPs), which were designed to target MMPs or thrombin, were tested in an animal model of atherothrombosis. ACPPs constitute a polycationic cell penetrating peptide (CPP) domain, a neutralizing polyanion and a cleavable linker to form a hairpin structure. The linker region was specifically designed so that protease dependent cleavage creates probe activation, releases CPP which allows the fluorescent-labeled CPP adherence in localized regions of elevated protease activity, thus providing a tool to generate an *in situ* spatial and focal protease activity map in diseased tissues [7,26]. We previously demonstrated the correlation of MMP and thrombin activated ACPPs uptake with plaque load and plaque severity in a murine model. However, the murine atherosclerosis does not mimic the stages of human plaques, and luminal thrombosis does not occur [7]. Furthermore, the challenge that must be met for future clinical applications is not simply detection of enzyme activity, which is present in all stages of atherogenesis [27], but the discrimination of individual high-risk plaques from stable plaques in vessels that often contain multiple plaques at various stages of risk.

The present study makes use of an established preclinical animal model (rabbit) of atherothrombosis that encompasses the above advantages for potential future translation to human disease and which simplifies the complicated plaque classification methods into a binominal model [14,15]. The novelty of this study is the combination of quantitative *in vivo* MRI of plaques and subsequent post-mortem fluorescence imaging of ACPPs to correlate disease state with localized protease activity in individual plaque. We hypothesized that these probes would show enhanced localized probe uptake in arterial plaques that are at high-risk for disruption, and the contribution of MRI-derived predictors would further complement the ACPPs in detecting disrupted plaques.

## Material and Methods

### Peptide synthesis and fluorophore labeling

Peptides were synthesized in house using standard protocols for Fmoc solid-phase synthesis and all peptides were amidated at their C-termini [26,28]. Peptides were N-terminally capped with either an acetyl or succinyl group on solid phase. Peptides were then labeled with Cy5 through a D-cysteine residue using Cy5-maleimide and standard coupling conditions. Specific peptide compositions were: thrombin cleavable peptide (DPRSFL–ACPP), Succinyl-(D-glutamyl)8-(5-amino-3-oxapentanoyl)-DPRSFL-(D-argininyl)9-Cy5-D-cysteinamide; MMP cleavable peptide (PLGLAG–ACPP),Acetyl-(D-glutamyl)9-(5-amino-3-oxapentanoyl)-PLGLAG-(D-argininyl)9-Cy5-D-cysteinamide; and control peptide (PEG-ACPP), Acetyl-(D- glutamyl)9-[NH(CH2CH2O)2CH2CO]2-(D-argininyl)9-Cy5-D-cysteinamide. All peptides were purified to greater than 95% purity using C–18 reverse phase HPLC with a 20–50% acetonitrile gradient in 0.1% TFA and the mass was confirmed by mass spectrometry [29].

### Animal model and timeline

**Fig 1:**
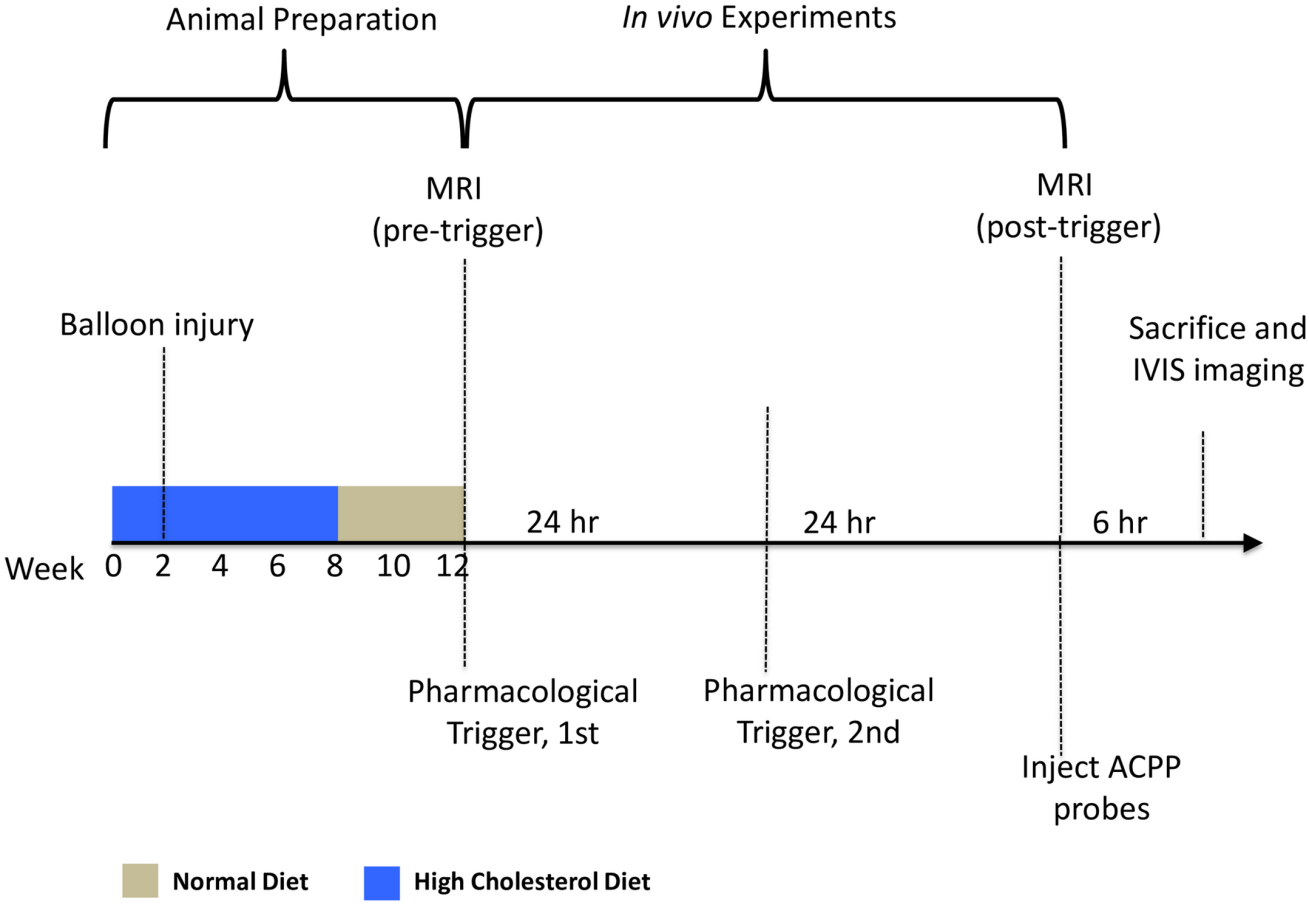
Timeline of the experiments. The animal preparation period took 12 weeks in total. Rabbits were fed on high cholesterol (1%) diet for continuous 8 weeks, followed by a 4-week normal diet treatment. The balloon surgery was given to rabbits two weeks into the high cholesterol diet to introduce endothelial denudation. After the animal preparation, pre- and post-trigger MRI scans were performed 48 hours apart prior and after two pharmacological triggerings. The ACPP probe was injected into rabbits intravenously after the 2^nd^ MRI section and allowed to circulate for 6 hours. The animals then were sacrificed and the fluorescent imaging was performed on extracted aortas.

Male New Zealand white (NZW) rabbits (N = 25) entered the study at 3 months old. Among which, 21 rabbits went through a 12-week atherogenic protocol, including 8 week high cholesterol diet (HCD, 1%), balloon surgery (incision side: right femoral artery), as previously described [14,30]. The remaining 4 rabbits (age, weight matched) were untreated, non-atherosclerotic control rabbits. After the 12-week diet treatment, thrombosis was induced via two pharmacological triggering sessions (Russell viper venom, 0.15 mg/kg IP; histamine, 0.02 mg/kg IV), which took a span of 48 hours in total. MRI of rabbit’s abdominal aorta was acquired both pre- and post- the triggering events [14,15]. After MRI, these rabbits were further divided into 5 experimental groups randomly (Table 1). Groups A (n = 9), B (n = 8) and C (n = 4) were atherosclerotic rabbits injected (IV) with 300 nmoles MMP-ACPP, thrombin-ACPP or PEG-ACPP respectively. Groups D-E (n = 2), included non-atherosclerotic rabbits, and was injected with the MMP-ACPP or thrombin-ACPP respectively. Because of the low tissue penetration depth of fluorescence signals, it was necessary to acquire images *ex vivo*. For contrast optimization purpose, probe was allowed to circulate for 6hrs. Animals were then sacrificed by pentobarbital (>120mg/Kg, IV) and aortas was excised and extended to physiological length and pinned down on a fluorescence free sample tray for optical imaging. Finally, transverse cryosections (10 μm) of aortas were collected and stained with Masson trichrome (Sigma Aldrich) to identify cellular components and thrombi. Meanwhile, at the end of the 6-hour ACPP circulation period, tissues from thoracic aorta, liver and muscle, were harvested, homogenized. The fluorescence readings of Cy5 were taken with 630nm (excitation) and 680nm (emission) for homogenized suspensions. The details and the relevant result were shown in the supporting data (S1 Appendix). The timeline of the study is summarized in Fig 1. This study was approved by the Institutional Animal Care and Use Committee (IACUC) of Boston University (protocol number: AN14245).

**Table 1:** Experimental groups.

Experimental Group	Diet	Balloon surgery	Fluorescence probe	Number of rabbits
A	1% cholesterol	Y	MMP-ACPP	9
B	1% cholesterol	Y	Thrombin-ACPP	8
C	1% cholesterol	Y	PEG-ACPP	4
D	Normal	N	MMP-ACPP	2
E	Normal	N	Thrombin-ACPP	2

### Imaging and histology alignment

Prior to actual MRI data acquisition, an ungated coronal 3D phase contrast MR angiogram was acquired as a scout image using a T1-weighted, fast-filed echo sequence. The left renal and iliac bifurcations on the scout image served as anatomic markers to plan the following MRI acquisition, and match with fluorescence imaging and histology (S2 Appendix).

### MRI data acquisition and analysis

*In vivo* MRI was using a 3 Tesla (3T) Intera Philips System (Cleveland, OH). Rabbits were sedated by acepromazine (0.75 mg/kg; IM), ketamine (35 mg/kg; IM) and xylazine (2.5 mg/kg; IM), and then placed in a 6-element knee coil in the supine position. The heart and respiration rate were carefully monitored during the whole MRI session. The pre- and post- trigger MRIs were performed using the same acquisition protocols. The ECG gated T1 black blood (T1BB) images with fat suppression were acquired in the transverse plane starting from the left renal bifurcation to the iliac bifurcation using the following parameters: slice thickness = 3mm, gap = 1mm, FOV = 120 x 125mm^2^, acquired matrix = 384x362, reconstructed resolution = 234μm^2^, TR = 2 cardiac cycles, TE = 10ms, acceleration factor = 15, flip angle = 90°, IR delay = 350ms, number of averages = 2, sense factor = 2, and a total scan time of about 7~8min. Then gadolinium chelate (Gd-DTPA, 0.1mmol/kg, Magnevist, Germany) was administrated intravenously before un-gated phase contrast MR angiography (PC-MRA) acquisition. The parameters for PC-MRA were: slice thickness = 2mm, gap = 1mm, FOV = 120x125mm^2^, TE = 7.4 ms, TR = 17ms, flip angle = 15°, number of averages = 2, Matrix = 128x122, acquired resolution = 977x984 μm^2^, reconstructed resolution = 190x190 μm^2^. Finally, post contrast-enhanced (CE) T1BB images were acquired 10~15 min after Gd-DTPA injection with parameters identical to the previously described T1BB imaging.

The pre-triggering MRI analysis was performed in ImageJ (NIH). The contours for the vessel area (VA) were manually traced slice-by-slice using the PC-MRA images. After correction for vessel tapering, the image slice with the least plaque area was chosen as a reference slice and the remodeling ratio (RR) was calculated [14] by VA_lesion_/VA_reference_. To quantify gadolinium uptake, the average intensity of the vessel wall of each slice was measured from the CE T1BB, and normalized to intensity measurement of the matched non-contrast enhanced T1BB images, which generates a Gd enhancement ratio (GdR). Post-trigger T1BB images were to help to register disrupted plaques, based on the presence or absence of mural thrombus, to following *ex vivo* fluorescence experiment.

### Fluorescence imaging and data analysis

The *ex vivo* aortas were first imaged in closed-view using an IVIS Spectrum imaging station (Caliper, Hopkinton, MA). 640nm excitation was used. The emission filter was tuned to 680nm and the exposure times were set between 0.25-2s. All comparisons within each figure are presented with the same exposure time. To match MRI slices, the fluorescence images of the closed-view aorta were segmented into continuous 4mm axial segments. Each segment was assigned as disrupted or non-disrupted based on visual inspection described later. The average fluorescence signal for each segment was obtained and normalized to signal from left femoral artery (without endothelial denudation/visually presented atherosclerosis) to obtain the fluorescence enhancement ratio (FER). To further clarify the major source of fluorescence signal at disrupted regions, aortas were opened longitudinally and then imaged (open-view) again with both thrombi attached and removed from the underlying plaque. During each optical image session, a reflective (photographic) image was acquired using the same geometric setting for alignment purpose. Regions of interest (ROIs) of the detached thrombi and surrounding plaques were manually traced, and signals within ROIs was averaged and normalized.

### Statistical analysis

Data are presented as mean ± SD. Comparisons between groups were performed using the unpaired student *t*-test. A two-tailed probability value of *p*< 0.05 was defined as statistically significant. The Receiver Operating Characteristic (ROC) curve was to assess the power of FER, RR and GdR in distinguishing disrupted vs. non-disrupted regions. The test accuracy was measured by the area under curve (AUC). An AUC<0.50 was defined as a test failure, and the larger the AUC the stronger the parameter’s detection power against plaque disruption. The integrated power of combining the above parameters in distinguishing plaque rupture was also evaluated. The GdR, RR and FER were first normalized to the scale of 0~10. The weighted association value was obtained using the equation: Σ_i_ *predictor*_*i*_ *weight*_*i*_. Where, *predictor*_*i*_ represents the normalized value of either GdR, RR or FER, and *weight*_*i*_ represents and percentage of contribution from the corresponding predictor with a range of 0~100% and an interval of 5%. The ROC analysis was performed for each combination of different weights. The weight combination with the largest AUC is considered as the optimized combined predictor.

### Disrupted plaque characterization

**Fig 2:**
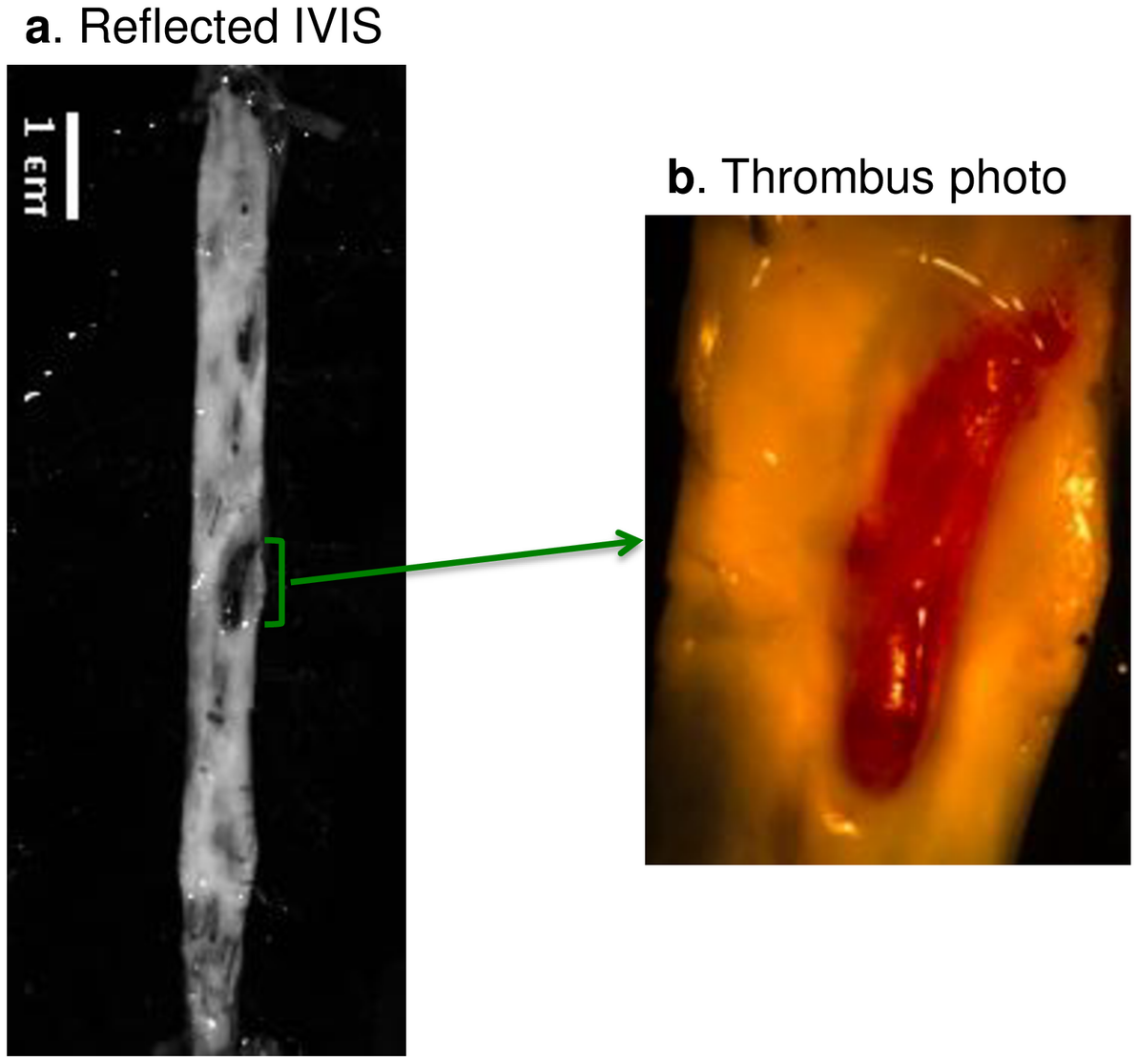
Identification of thrombus. The aorta was cut open longitudinally to reveal the internal structure. One dark region (green bracket) on the reflected image (a) represents an attached thrombus. However, the dark region in the reflected images is a not reliable criterion as white-light visual inspection, since dark regions may also indicate clotted blood and or normal vascular wall without presence of fatty streaks. The gold standard to verify the presence of a thrombus is visualization by naked eye in the excised aorta, shown as the example light-microscopy photo (b).

The presence or absence of attached thrombus could be identified and documented by white-light visual inspection when opening the aorta (Fig 2). Segments with a mural thrombus attached were defined as disrupted, and the ones without as stable. This was used as the gold standard to distinguish disrupted and non-disrupted plaques.

## Results

Atherosclerotic plaques were observed in all experimental rabbits on the high cholesterol diet (Groups: A-C). Nine atherosclerotic rabbits were studied with the MMP-ACPP, and 8 with the thrombin-ACPP probe; a total of 47 segments encompassing disrupted regions and 239 segments in non-disrupted regions were analyzed quantitatively.

### Fluorescence images

**Fig 3:**
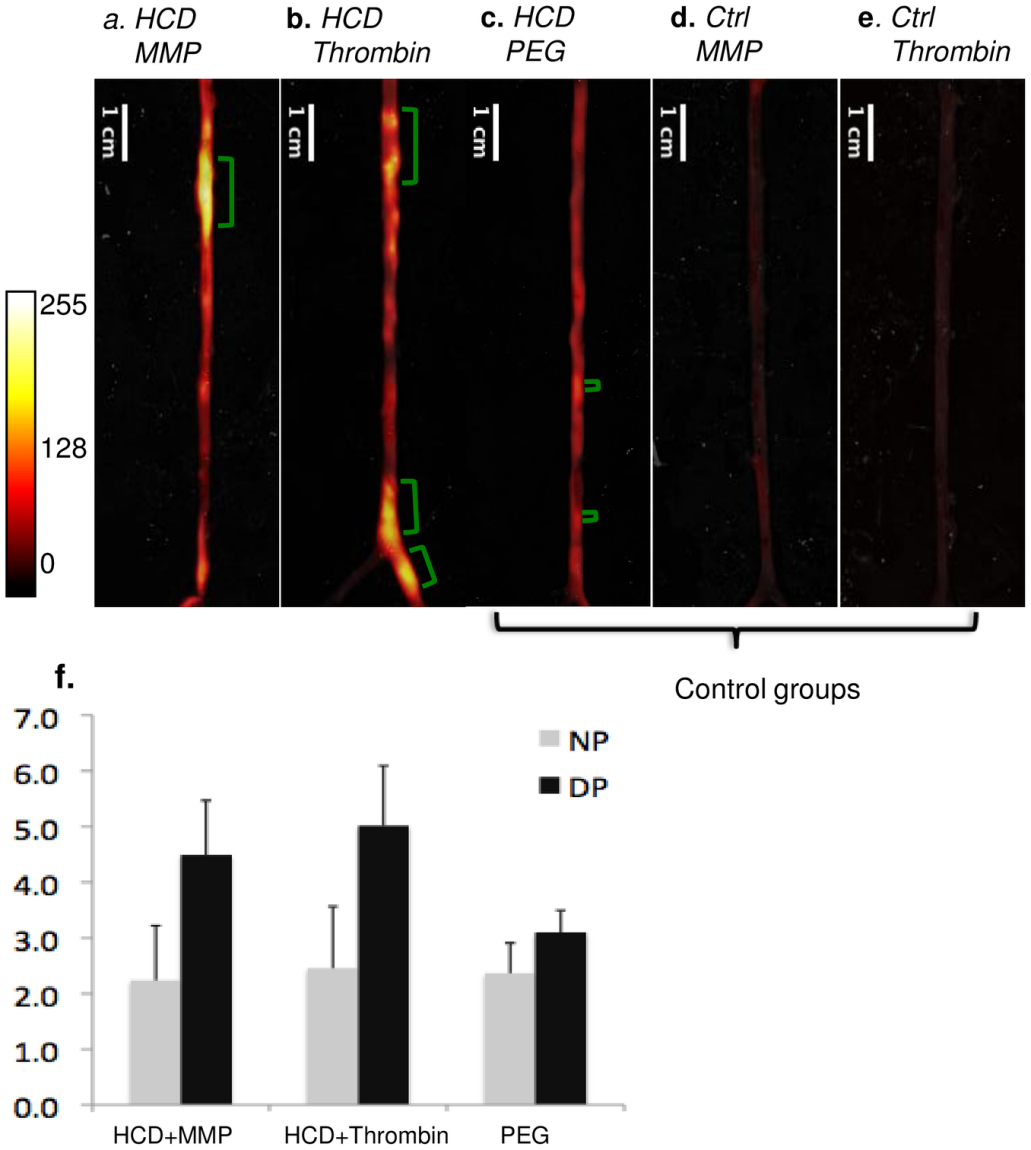
Representative *ex-vivo* fluorescence images of rabbits in 5 experimental groups. group A, HCD + MMP-ACPP(a); group B, HCD + Thrombin-ACPP (b); group C, HCD + PEG-ACPP(c); group D, normal diet + MMP-ACPP(d); normal diet + Thrombin-ACPP(e). Note that only one probe is injected into an animal, and all figures are from different animals. Within each experimental group, the closed view fluorescent images are presented with overlaid reflected images. The locations of thrombi are indicated by green brackets. Two-tailed t-test showed that fluorescence of ACPP probes was significantly higher in disrupted regions (DP) (p<0.001) compared to non-disrupted plaque regions (NP), whereas the non-cleavable PEG-ACPP had similar uptake into both regions (f).

Representative fluorescence images (closed-view) of rabbit aortas are shown in Fig 3a–3e. MMP or thrombin targeted ACPPs (Fig 3a and 3b) experienced substantial increased uptake in atherosclerotic regions, as compared to the three control groups: two groups of non-atherosclerotic rabbits injected with either targeted ACPPs (Fig 3d and 3e), and one group with atherosclerotic rabbits with control uncleavable PEG-ACPP (Fig 3c). To explore whether the targeted probes would accumulate excessively in regions at high-risk region, the plaques were further divided into disrupted and non-disrupted groups. At disrupted region, the targeted ACPPs (Fig 3f) showed significant (all p<0.001) signal increase (MMP/thrombin probe: 4.5±1.0/5.0±1.1) as compared to non-disrupted areas (MMP/thrombin probe: 2.2±1.0/2.5±1.1); in contrast, similar signal enhancement was not observed for the control PEG probe.

**Fig 4:**
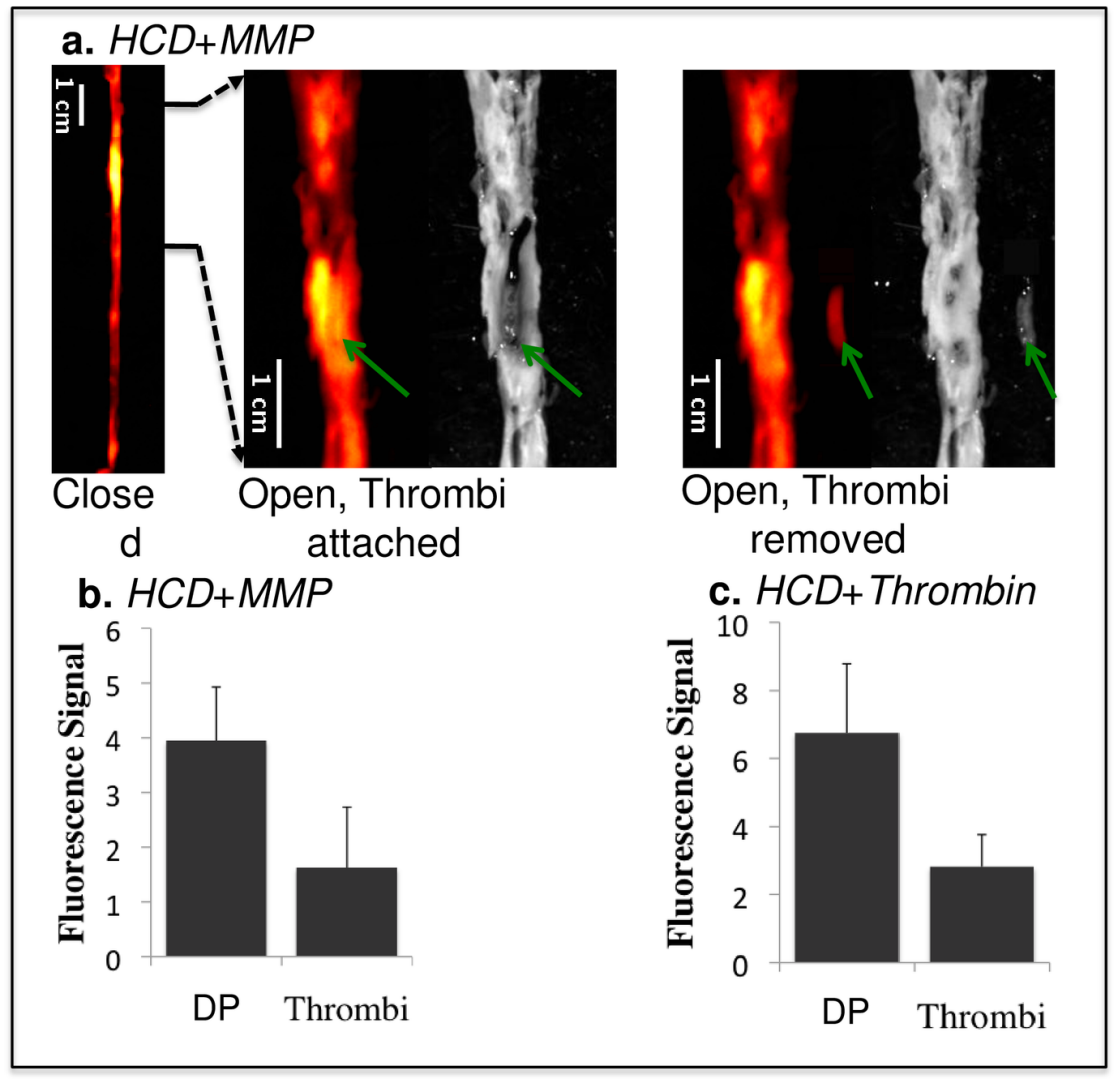
Comparison of fluorescence signals from thrombi and the surrounding atherosclerotic plaque in the experimental group. HCD + MMP-ACPP (a,b) and HCD + Thrombin-ACPP(c). The images from left to right in **a** are: closed view fluorescence image, opened and zoomed-in fluorescence/reflected images with the thrombus (green arrows) attached at its original site, opened and zoomed-in fluorescence/reflected images with the thrombus removed. The dark signal of the aorta on the reflected image comes from clotted blood and relatively healthy vessel wall (without obvious fatty streaks). Plaques appeared as brighter (gray-ish) signal. The bar graphs (b,c) give the statistical analysis of fluorescence signal for the underlying disrupted plaques (DP) and the overlaying thrombi (p<0.001).

To examine whether the high fluorescence signal at the sites of plaque thrombosis originated from the plaque or the thrombus, the aortas were opened longitudinally and the thrombi removed from their attachment sites. As illustrated for the MMP-probe in Fig 4a, the greatest enhancement originated from the underlying plaque rather than from the attached thrombus. This was confirmed by statistical analysis of data for both probes in all rabbits (Fig 4b and 4c). In the longitudinally opened images, the underlying plaques showed a 4.0±1.1 signal increase for the MMP probe and an even higher increase (6.8±2.0) for the thrombin probe.

### Correspondence between fluorescence and MRI

**Fig 5:**
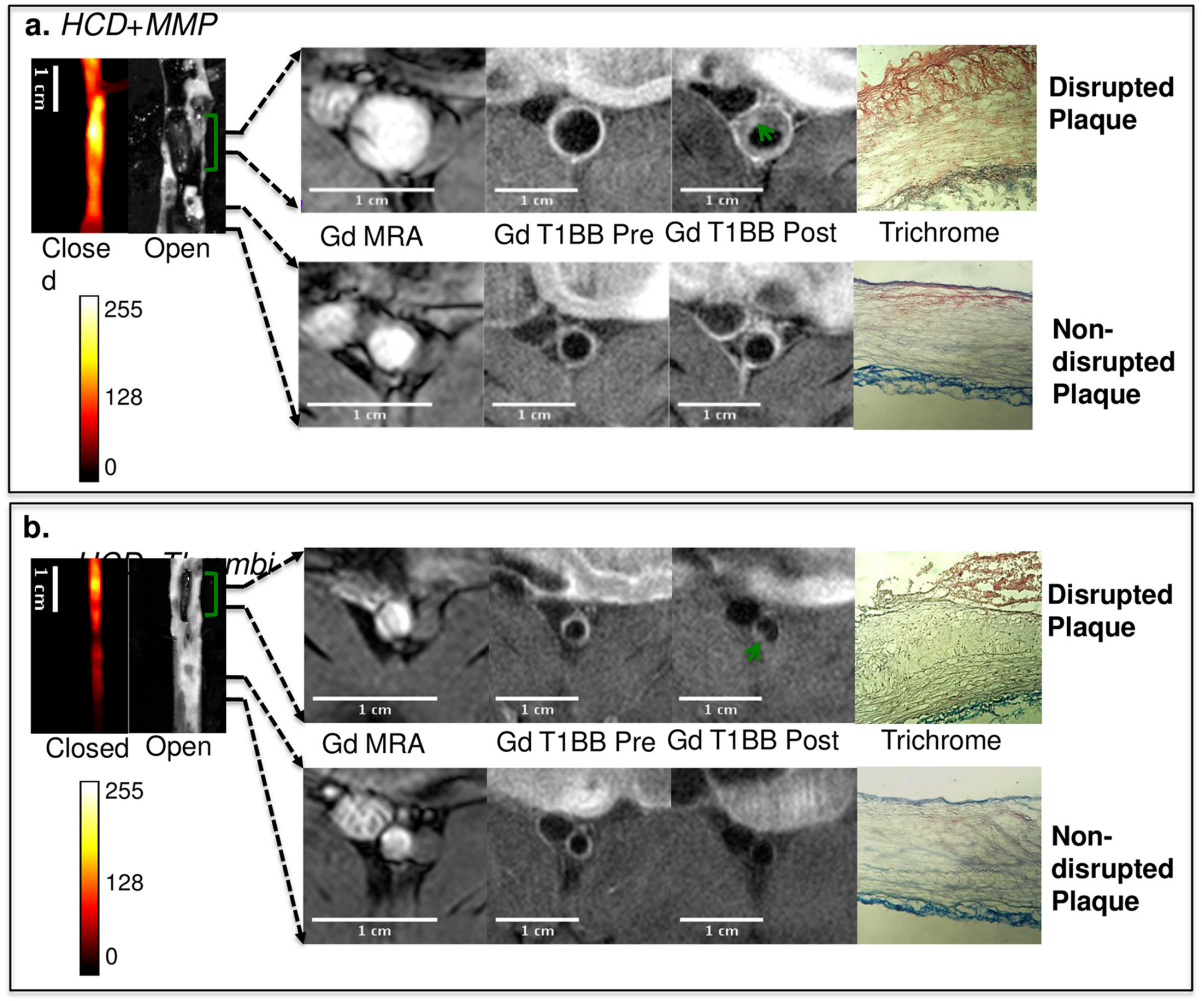
Representative fluorescence images and co-registered MRI images for rabbits from group A: HCD + MMP-ACPP(a) and group B: HCD + Thrombin-ACPP(b). The closed view of the fluorescence images shows a sub-section of abdominal aorta and the corresponding open view reflected images showing the location of thrombi (green brackets). The regions within the two black lines represent examples of non-disrupted and disrupted plaques respectively, and correspond to the MRI images on the right. All MRI images were obtained after gadolinium-DTPA injection according to our acquisition protocols; illustrated are the pre-triggering MRA and the fat-suppressed T1BB images obtained before and after pharmacological triggering (from left to right). Thrombi are marked by green arrows in post-triggering Gd-T1BB (T1 black blood) images. With the current resolution of MRI, it is very difficult to distinguish different layers of plaque. Hence, in the post-triggering MRI, the formed thrombus, which is on a relatively macro-scale, instead of plaque details, was observed. The corresponding histological images (after the thrombus was removed) with trichrome staining were presented on the very right column.

To explore whether or not MRI derived predicators are complementary to ACPP measurements in disrupted regions, the uptake of gadolinium chelate and outward remodeling from *in vivo* MRI images and corresponding ex-vivo measured fluorescent signal were analyzed together. Fig 5 shows examples of MRI images representative of non-disrupted (Fig 5a and 5b, bottom row) and disrupted (Fig 5a and 5b, upper row) plaques co-registered with the corresponding fluorescence and histological images of the vessels. Both disrupted regions were accompanied by higher Gd-DTPA uptake than the non-disrupted plaques in the pre-trigger image. However, only one of the presented disrupted regions showed excessive outward remodeling (Fig 5a). The discrepancy between ACPP and MRI- derived measures suggest that the uptake of ACPPs may represent a different biological process as compared to remodeling and Gd-DTPA uptake. Thus the ACPPs and the MRI measures might complement each other, and the combination of the two might provide more predictive power than each method alone. To address this issue, Receiver Operating Characteristic (ROC) analysis was performed for the following factors, including FER, GdR and RR, both alone and combined.

**Fig 6:**
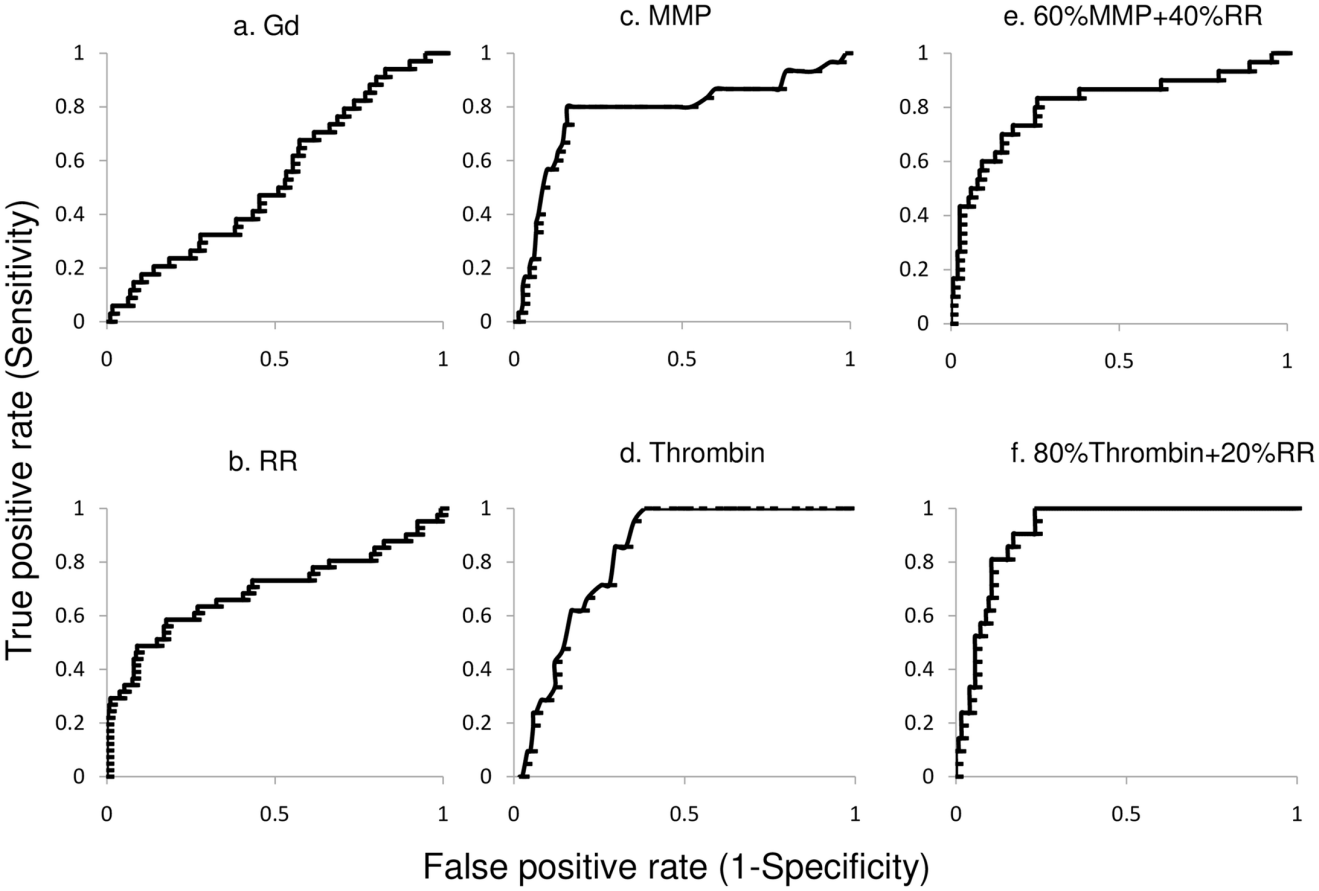
ROC analyses. The Gd-DTPA uptake did not show a strong prediction for plaque rupture, with AUC = 0.51±0.05 (a). The remodeling-ratio (RR) showed an AUC of 0.69±0.05 (b). Its optimal cutoff was 1.06, with a corresponding specificity of 84.2% and sensitivity of 58.5%. The area under the curve (AUC) was 0.79±0.05 for the MMP-ACPP (c) and 0.90±0.05 for the Thrombin-ACPP probe (d). The optimal cutoff threshold was 3.5/4.0 fold (MMP-ACPP/Thrombin-ACPP), with corresponding specificities of 84.2%/ 83.2% and sensitivities of 80.0%/ 85.7%. The detection power for plaque disruption was further increased when both MRI remodeling ratio and ACPP uptake was considered together. For the MMP-ACPP, the maximum AUC (0.81±0.05) was achieved when the combined predictor had contributions comprised of 60% ACPP uptake and 40% remodeling ratio (e). Similarly, for the thrombin probe the maximum AUC (0.92±0.05) was achieved when the combined predictor contributed by 80% ACPP uptake and 20% remodeling ratio (f).

When comparing each single factor, the targeted ACPPs demonstrated superior detection power. The AUC was 0.79±0.05 for the MMP probe and even higher (0.90±0.05) for the thrombin probe. The optimal cutoff threshold was 3.5/4.0 fold (MMP / thrombin), with corresponding specificities of 84.2%/ 83.2% (MMP / thrombin) and sensitivities of 80.0%/ 85.7% (MMP / thrombin). The remodeling ratio showed a smaller AUC (0.69±0.05) and the GdR showed an even smaller AUC (0.51±0.05, Fig 6). The detection power for plaque disruption was further increased when both MRI remodeling ratio and ACPP uptake was considered together. For the MMP-ACPP, the maximum AUC (0.81±0.05) was achieved when the combined predictor had contributions comprised of 60% ACPP uptake and 40% remodeling ratio. Similarly, for the thrombin probe the maximum AUC (0.92±0.05) was achieved when the combined predictor contributed by 80% ACPP uptake and 20% remodeling ratio. Including the GdR in the model did not generate better predictions. The results for different predictors were summarized in Table 2.

**Table 2:** Comparison of ROC results.

	AUC	Sensitivity	Specificity
MMP-ACPP (FER)	0.79	80.0%	84.2%
Thrombin-ACPP (FER)	0.90	85.7%	83.2%
Remodeling ratio (RR)	0.69	58.5%	84.2%
Gd-DTPA (GdR)	0.51	NA	NA
Combined: MMP-ACPP (FER): 60% RR: 40%	0.81	80.2%	81.0%
Combined:Thrombin-ACPP (FER): 80%RR: 20%	0.92	90.5%	83.2%

## Discussion

In this study, we show that two novel, targeted fluorescent ACPPs detected disrupted plaques with high sensitivity and specificity in a rabbit model of accelerated atherosclerosis. As with any animal model, the rabbit model used here cannot completely mimic atherosclerotic disease progression found in humans. However, this rabbit model develops 6 of the 8 AHA categories for human plaques, and the disrupted plaques shared several histological features known to be associated with vulnerable plaques in humans [30,31]. Our model uses the pro-coagulant factor (viper venom) and a vasoconstriction agent (histamine) as plaque rupture catalysts. Although the exact mechanism for plaque rupture in humans has not been identified, platelet activation and adhesion and the release of vasoconstriction molecules are possible critical steps in the process [32]. The induced thrombus is platelet rich with fibrin and collagen [33], and has been shown to replicate features of human thrombosis including tissue factor expression and its regulation by NF-kB [34,35]. This rabbit model provides a method for studying thrombosis in a time-controlled manner and enables objective testing of protease cleavable probes for identifying high-risk plaques by providing a simplified, yet effective binominal plaque classification (disrupted *vs*. non-disrupted), rather than broad and complicated histological features of vulnerability.

Currently, various imaging methods have made advances in detecting “high-risk” plaques among stable ones in human vascular disease. In addition to traditional technology, such as computed tomography (CT) or ultrasound, which focuses on occlusion of lumen, emerging methods have started to concentrate on functional aspects of atherosclerosis. For example, fractional flow reserve (FFR) can provide valuable information about the attainable blood flow despite stenosis [36]. Positron emission tomography (PET) employing fluorine-18-labeled 2-deoxy-D-glucose (FDG), can reveal metabolic activity in advanced plaques [37]. Among these technologies, targeted ACPPs have unique advantages. The uptake of ACPP can reflect the level of the specific enzyme, which may directly participate in plaque destabilization. Its special uptake mechanism can effectively amplify the signal locally. This type of imaging provides an activity-based visualization of plaques, which can be complementary to the morphological information typically obtained via imaging. In this study, both MMP and thrombin-cleavable ACPPs appear to distinguish between disrupted and non-disrupted plaques. However, we have previously noted that only thrombin-cleavable and not MMP-cleavable ACPP correlated with plaque severity in the ApoE-/- and LDLR-/- mice [7]. This difference likely reflects variability between animal models, as our rodent models did not produce “disrupted plaques”. There is some background uptake of the non-targeted control probe (PEG-ACPP) in atherosclerotic regions (Fig 3c) which may be due to incomplete blocking of the CPP domain by the inhibitory domain with the ACPP [7,28,38].

A crucial strategy of our study was to combine ACPP measurements and MRI derived predictors for plaque disruption, the pathophysiological end point of high-risk plaques. MRI derived measures, outward remodeling and enhanced gadolinium-DTPA uptake, have been linked to plaque destabilization in previous studies [14,39,40]. However, the calculation of RR has limitations imposed by the necessity for mathematical modeling of the often complex geometry of the vessel. Although the Gd-DTPA often shows uptake in vulnerable plaques, passive diffusion of the contrast agent into stable plaques often results in a high false positive rate. Our data showed the MRI derived measures are inferior (AUC = 0.51–0.69) yet may complement ACPPs in detecting disrupted plaques. A larger AUC value is achieved by combining contributions from both the ACPP and the remodeling ratio. However, due to passive diffusion being a common occurrence shared by both Gd-DTPA and ACPPs, Gd-DTPA did not provide additional information and thus cannot be used to improve the detecting power of the ACPPs.

The main limitation of using the Cy5 labeled ACPPs reported here is the necessity to remove and expose the aorta for fluorescence imaging due to the relatively low penetration depth of transmitted light. In addition, the probe contrast reaches its maximum at about the 6th hour, and the animal must then be sacrificed for imaging. This makes fluorescent imaging with a 48-hour triggering period incompatible with probe injection prior to triggering rupture. For future clinical applications, this limitation could be obviated by catheter-based imaging [41] or possibly by imaging from outside the vessel wall during surgery. Both methods may have potentials in pre-clinical settings, however, neither is currently used clinically, and such methods would be quite invasive. It would be more desirable to use ACPPs to molecularly target MRI, as it allows for minimally invasive, *in vivo* applications, and therefore, a higher likelihood for translational development. Additionally, MRI would permit the detection of probe uptake in a pixel-by-pixel manner. This feature could clarify the potential ambiguity lying in the current optic imaging setting for the source of signal increase: from elevated enzyme density, which indicates plaque destabilization, or merely from thickening of vessel wall, which is observed more often in stable plaques. Furthermore, with remodeling ratio and ACPP uptake that can be measured in the same MRI session, any systematic error caused by matching different imaging modality will be eliminated. A challenge for molecularly targeted MRI probes to overcome is the limitation of low sensitivity that has been achieved in studies with other probe designs [42,43]. The design flexibility of the ACPPs has permitted delivery of detectable levels of Gd in tumors for MRI imaging [44]. In another technical advance, we identified and reported [38] an improved thrombin substrate, with the sequence Nle-TPRSFL, that increased the k_cat_/K_m_ for ACPP cleavage by thrombin by 55 fold in a mouse model. This increased enzyme sensitivity should lower the amount of probe needed, which in turn may lower the potentially toxicity, and facilitate translation into the rabbit model for non-invasive MRI detection of destabilized plaques. Future development of gadolinium-loaded ACPPs for the MMPs and thrombin would allow detection of enzymatic activity and vascular remodeling in a single MRI examination and could significantly enhance *in vivo* diagnosis of vulnerable plaques at high risk for disruption.

## Supporting Information

S1 Appendix ACPP uptake in other organs. (DOCX)

S2 Appendix MR-angiogram for planning MRI scans and co-register with fluorescence images and histology. (DOCX)

## References

[1] Roger VL, Go AS, Lloyd-Jones DM, Adams RJ, Berry JD, Brown TM, et al Heart disease and stroke statistics–-2011 update: a report from the American Heart Association. Circulation. 2011 2 1;123(4):e18–e209. 10.1161/CIR.0b013e3182009701 21160056 PMC4418670

[2] Libby P. Inflammation in atherosclerosis. Arterioscler Thromb Vasc Biol. 2012 9;32(9):2045–51. 10.1161/ATVBAHA.108.179705 22895665 PMC3422754

[3] Lusis AJ. Atherosclerosis. Nature. 2000 9 14;407(6801):233–41. 11001066 10.1038/35025203 PMC2826222

[4] Makowski MR, Wiethoff AJ, Blume U, Cuello F, Warley A, Jansen CHP, et al Assessment of atherosclerotic plaque burden with an elastin-specific magnetic resonance contrast agent. Nat Med. 2011 3;17(3):383–8. 10.1038/nm.2310 21336283

[5] Amirbekian V, Lipinski MJ, Briley-Saebo KC, Amirbekian S, Aguinaldo JGS, Weinreb DB, et al Detecting and assessing macrophages in vivo to evaluate atherosclerosis noninvasively using molecular MRI. Proc Natl Acad Sci USA. 2007 1 16;104(3):961–6. 17215360 10.1073/pnas.0606281104 PMC1766334

[6] Cai K, Caruthers SD, Huang W, Williams TA, Zhang H, Wickline SA, et al MR molecular imaging of aortic angiogenesis. JACC Cardiovasc Imaging. 2010 8;3(8):824–32. 10.1016/j.jcmg.2010.03.012 20705262 PMC3425389

[7] Olson ES, Whitney MA, Friedman B, Aguilera TA, Crisp JL, Baik FM, et al In vivo fluorescence imaging of atherosclerotic plaques with activatable cell-penetrating peptides targeting thrombin activity. Integr Biol (Camb). 2012 6;4(6):595–605. 22534729 10.1039/c2ib00161f PMC3689578

[8] Lancelot E, Amirbekian V, Brigger I, Raynaud J-S, Ballet S, David C, et al Evaluation of matrix metalloproteinases in atherosclerosis using a novel noninvasive imaging approach. Arterioscler Thromb Vasc Biol. 2008 3;28(3):425–32. 10.1161/ATVBAHA.107.149666 18258820

[9] Temma T, Ogawa Y, Kuge Y, Ishino S, Takai N, Nishigori K, et al Tissue factor detection for selectively discriminating unstable plaques in an atherosclerotic rabbit model. J Nucl Med. 2010 12;51(12):1979–86. 10.2967/jnumed.110.081216 21078793

[10] Fayad ZA, Fuster V. Clinical imaging of the high-risk or vulnerable atherosclerotic plaque. Circ Res. 2001 8 17;89(4):305–16. 11509446 10.1161/hh1601.095596

[11] Choudhury RP, Fuster V, Badimon JJ, Fisher EA, Fayad ZA. MRI and characterization of atherosclerotic plaque: emerging applications and molecular imaging. Arterioscler Thromb Vasc Biol. 2002 7 1;22(7):1065–74. 12117718 10.1161/01.atv.0000019735.54479.2f

[12] Yuan C, Hatsukami TS, O'Brien KD. High-Resolution magnetic resonance imaging of normal and atherosclerotic human coronary arteries ex vivo: discrimination of plaque tissue components. J Investig Med. 2001 11;49(6):491–9. 11730084 10.2310/6650.2001.33625

[13] Kim WY, Stuber M, Börnert P, Kissinger KV, Manning WJ, Botnar RM. Three-dimensional black-blood cardiac magnetic resonance coronary vessel wall imaging detects positive arterial remodeling in patients with nonsignificant coronary artery disease. Circulation. 2002 7 16;106(3):296–9. 12119242 10.1161/01.cir.0000025629.85631.1e

[14] Phinikaridou A, Ruberg FL, Hallock KJ, Qiao Y, Hua N, Viereck J, et al In vivo detection of vulnerable atherosclerotic plaque by MRI in a rabbit model. Circ Cardiovasc Imaging. 2010 5;3(3):323–32. 10.1161/CIRCIMAGING.109.918524 20194634

[15] Phinikaridou A, Hua N, Pham T, Hamilton JA. Regions of Low Endothelial Shear Stress Co-localize with Positive Vascular Remodeling and Atherosclerotic Plaque Disruption: An in vivo MRI Study. Circ Cardiovasc Imaging. 2013 1 28. 10.1161/CIRCIMAGING.112.000176 23357244

[16] Moreno PR, Purushothaman K-R, Sirol M, Levy AP, Fuster V. Neovascularization in human atherosclerosis. Circulation. 2006 5 9;113(18):2245–52. 16684874 10.1161/CIRCULATIONAHA.105.578955

[17] Galis ZS, Sukhova GK, Lark MW, Libby P. Increased expression of matrix metalloproteinases and matrix degrading activity in vulnerable regions of human atherosclerotic plaques. J Clin Invest. 1994 12;94(6):2493–503. 7989608 10.1172/JCI117619 PMC330083

[18] Galis ZS, Khatri JJ. Matrix metalloproteinases in vascular remodeling and atherogenesis: the good, the bad, and the ugly. Circ Res. 2002 2 22;90(3):251–62. 11861412

[19] Chatzizisis YS, Baker AB, Sukhova GK, Koskinas KC, Papafaklis MI, Beigel R, et al Augmented expression and activity of extracellular matrix-degrading enzymes in regions of low endothelial shear stress colocalize with coronary atheromata with thin fibrous caps in pigs. Circulation. 2011 2 15;123(6):621–30. 10.1161/CIRCULATIONAHA.110.970038 21282495 PMC3066078

[20] Choudhary S, Higgins CL, Chen IY, Reardon M, Lawrie G, Vick GW, et al Quantitation and localization of matrix metalloproteinases and their inhibitors in human carotid endarterectomy tissues. Arterioscler Thromb Vasc Biol. 2006 10;26(10):2351–8. 16888239 10.1161/01.ATV.0000239461.87113.0b

[21] Scholtes VPW, Johnson JL, Jenkins N, Sala-Newby GB, de Vries J-PPM, de Borst GJ, et al Carotid atherosclerotic plaque matrix metalloproteinase-12-positive macrophage subpopulation predicts adverse outcome after endarterectomy. J Am Heart Assoc. 2012 12;1(6):e001040 10.1161/JAHA.112.001040 23316311 PMC3540663

[22] Borissoff JI, Spronk HMH, Heeneman S, Cate ten H. Is thrombin a key player in the “coagulation-atherogenesis” maze? Cardiovasc Res. 2009 6 1;82(3):392–403. 10.1093/cvr/cvp066 19228706

[23] Byrne CD, Wild SH. The Metabolic Syndrome. Wiley-Blackwell; 2011 1 p.

[24] Galis ZS, Kranzhöfer R, Fenton JW, Libby P. Thrombin promotes activation of matrix metalloproteinase–2 produced by cultured vascular smooth muscle cells. Arterioscler Thromb Vasc Biol. 1997 3;17(3):483–9. 9102166 10.1161/01.atv.17.3.483

[25] Koo BH, Han JH, Yeom YI, Kim DS. Thrombin-dependent MMP–2 activity is regulated by heparan sulfate. Journal of Biological Chemistry. 2010. 10.1074/jbc.M110.171595 PMC3009852 21041295

[26] Jiang T, Olson ES, Nguyen QT, Roy M, Jennings PA, Tsien RY. Tumor imaging by means of proteolytic activation of cell-penetrating peptides. Proc Natl Acad Sci USA. 2004 12 21;101(51):17867–72. 15601762 10.1073/pnas.0408191101 PMC539314

[27] Razavian M, Tavakoli S, Zhang J, Nie L, Dobrucki LW, Sinusas AJ, et al Atherosclerosis plaque heterogeneity and response to therapy detected by in vivo molecular imaging of matrix metalloproteinase activation. J Nucl Med. 2011 11;52(11):1795–802. 10.2967/jnumed.111.092379 21969358 PMC3235922

[28] Olson ES, Aguilera TA, Jiang T, Ellies LG, Nguyen QT, Wong EH, et al In vivo characterization of activatable cell penetrating peptides for targeting protease activity in cancer. Integr Biol (Camb). 2009 6;1(5–6):382–93. 20023745 10.1039/b904890a PMC2796841

[29] Whitney M, Crisp JL, Olson ES, Aguilera TA, Gross LA, Ellies LG, et al Parallel in vivo and in vitro selection using phage display identifies protease-dependent tumor-targeting peptides. J Biol Chem. 2010 7 16;285(29):22532–41. 10.1074/jbc.M110.138297 20460372 PMC2903386

[30] Phinikaridou A, Hallock KJ, Qiao Y, Hamilton JA. A robust rabbit model of human atherosclerosis and atherothrombosis. J Lipid Res. 2009 5 1;50(5):787–97. 10.1194/jlr.M800460-JLR200 19141434 PMC2666165

[31] Pasterkamp G, Borst C, Gussenhoven EJ, Mali WP, Post MJ, The SH, et al Remodeling of De Novo atherosclerotic lesions in femoral arteries: impact on mechanism of balloon angioplasty. J Am Coll Cardiol. 1995 8;26(2):422–8. 7608445 10.1016/0735-1097(95)80017-b

[32] Saam T, Underhill HR, Chu B, Takaya N, Cai J, Polissar NL, et al Prevalence of American Heart Association type VI carotid atherosclerotic lesions identified by magnetic resonance imaging for different levels of stenosis as measured by duplex ultrasound. J Am Coll Cardiol. 2008 3 11;51(10):1014–21. 10.1016/j.jacc.2007.10.054 18325441

[33] Phinikaridou A, Qiao Y, Giordano N, Hamilton JA. Detection of thrombus size and protein content by ex vivo magnetization transfer and diffusion weighted MRI. J Cardiovasc Magn Reson. 2012;14:45 10.1186/1532-429X-14-45 22731842 PMC3419091

[34] Yamashita A, Zhao Y, Zhao S, Matsuura Y, Sugita C, Iwakiri T, et al Arterial (18)F-fluorodeoxyglucose uptake reflects balloon catheter-induced thrombus formation and tissue factor expression via nuclear factor-κB in rabbit atherosclerotic lesions. Circ J. 2013;77(10):2626–35. 23832535 10.1253/circj.cj-12-1463

[35] Yamashita A, Asada Y. A rabbit model of thrombosis on atherosclerotic lesions. J Biomed Biotechnol. 2011;2011:424929 10.1155/2011/424929 21253503 PMC3021877

[36] Pijls NH, Van Gelder B, Van der Voort P, Peels K, Bracke FA, Bonnier HJ, et al Fractional flow reserve. A useful index to evaluate the influence of an epicardial coronary stenosis on myocardial blood flow. Circulation. 1995 12 1;92(11):3183–93. 7586302 10.1161/01.cir.92.11.3183

[37] Tarkin JM, Joshi FR, Rudd JHF. PET imaging of inflammation in atherosclerosis. Nat Rev Cardiol. Nature Publishing Group; 2014 8 1;11(8):443–57. 10.1038/nrcardio.2014.80 24913061

[38] Whitney M, Savariar EN, Friedman B, Levin RA, Crisp JL, Glasgow HL, et al Ratiometric activatable cell-penetrating peptides provide rapid in vivo readout of thrombin activation. Angew Chem Int Ed Engl. 2013 1 2;52(1):325–30. 10.1002/anie.201205721 23080482 PMC3694763

[39] Schoenhagen P, Ziada KM, Kapadia SR, Crowe TD, Nissen SE, Tuzcu EM. Extent and direction of arterial remodeling in stable versus unstable coronary syndromes: an intravascular ultrasound study. Circulation. 2000 2 15;101(6):598–603. 10673250 10.1161/01.cir.101.6.598

[40] Yeon SB, Sabir A, Clouse M, Martinezclark PO, Peters DC, Hauser TH, et al Delayed-enhancement cardiovascular magnetic resonance coronary artery wall imaging: comparison with multislice computed tomography and quantitative coronary angiography. J Am Coll Cardiol. 2007 7 31;50(5):441–7. 17662397 10.1016/j.jacc.2007.03.052

[41] Yoo H, Kim JW, Shishkov M, Namati E, Morse T, Shubochkin R, et al Intra-arterial catheter for simultaneous microstructural and molecular imaging in vivo. Nat Med. 2011 12;17(12):1680–4. 10.1038/nm.2555 22057345 PMC3233646

[42] Jaffer FA, Kim D-E, Quinti L, Tung C-H, Aikawa E, Pande AN, et al Optical visualization of cathepsin K activity in atherosclerosis with a novel, protease-activatable fluorescence sensor. Circulation. 2007 5 1;115(17):2292–8. 17420353 10.1161/CIRCULATIONAHA.106.660340

[43] Li Z, Li L, Zielke HR, Cheng L, Xiao R, Crow MT, et al Increased expression of 72-kd type IV collagenase (MMP–2) in human aortic atherosclerotic lesions. Am J Pathol. 1996 1;148(1):121–8. 8546199 PMC1861591

[44] Olson ES, Jiang T, Aguilera TA, Nguyen QT, Ellies LG, Scadeng M, et al Activatable cell penetrating peptides linked to nanoparticles as dual probes for in vivo fluorescence and MR imaging of proteases. Proc Natl Acad Sci USA. 2010 3 2;107(9):4311–6. 10.1073/pnas.0910283107 20160077 PMC2840175

