# Supplementary material for: Identification of High-Risk Plaques by MRI and Fluorescence Imaging in a Rabbit Model of Atherothrombosis
Source: PLoS One. 2015 Oct 8;10(10):e0139833. doi: 10.1371/journal.pone.0139833 (PMC4598148; doi:10.1371/journal.pone.0139833)
Supplement: S1 Appendix — (DOCX) [file pone.0139833.s001.docx]

**S1 Appendix: ACPP uptake in other organs.**

At the end of the 6-hour ACPP circulation period, tissue was harvested, homogenized and suspended in a plate reader. Aorta segments were obtained from levels known to have little vulnerable plaque, as these are distal to the site of balloon injury. In parallel, liver and muscle samples harvested. Tissues were weighed and homogenized in 9x volumes of a SDS homogenization buffer (25mg/ml ProK, 0.1mg/ml DNAse, 150mM NaCl, 10mM TRIS PH8, 0.2% SDS) formulated to preserves Cy5 fluorescence. After overnight incubation at 37 degrees, the tissue was sonicated. Equivalent volumes were pipetted into 96 well clear bottom plates. The fluorescence readings for Cy5 were taken with 630nm (excitation) and 680nm (emission).

The data showed relatively low fluorescence in aorta homogenates (Fig. S1). There was no difference between fluorescence in aorta homogenates from rabbits injected with the thrombin-cleavable, or MMP-cleavable or uncleavable (PEG) ACPPs. This indicates ACPP aorta uptake was equivalent and that local differences in signal are not due to gross differences in uptake. The uptake of ACPPs in muscle is relatively low as compared to aorta uptake. Consistent with a hepatic route of ACPP clearance, there was significantly higher fluorescence present in liver homogenates as compared to aortas/muscles from the same rabbit (p<0.05, two tailed t-test). However, there was no significant difference in liver uptake of thrombin-cleavable, MMP-cleavable or uncleavable ACPPs. This further establishes that each rabbit was dosed with a consistent dose of Cy5-labelled ACPP.


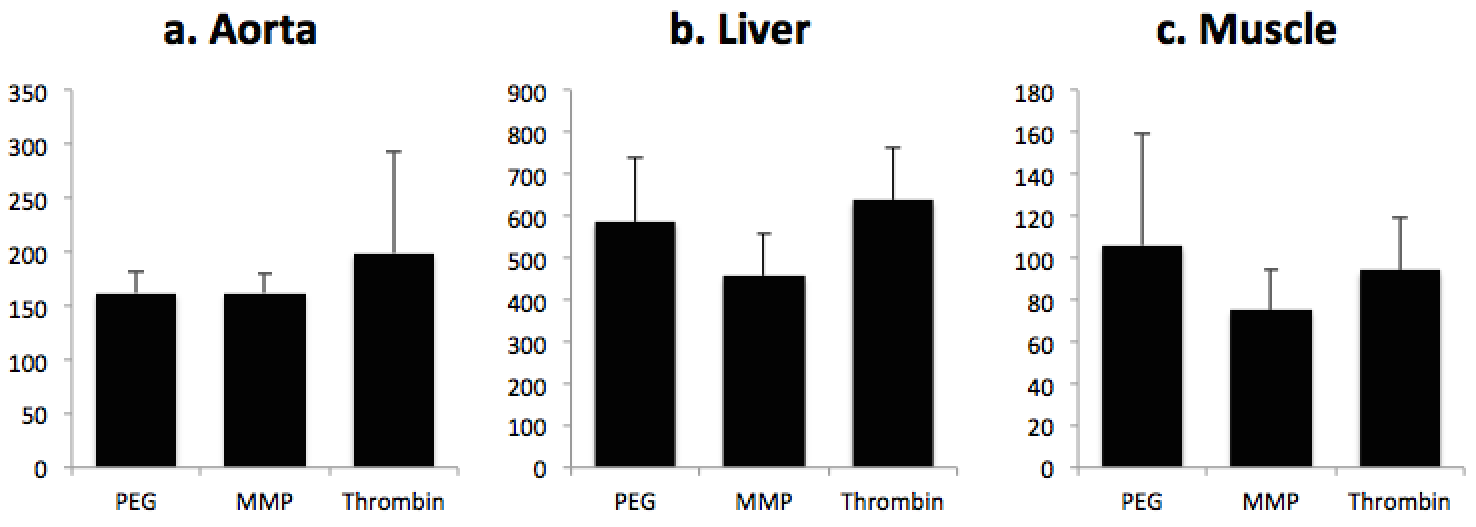


Figure S1: The comparison of MMP-cleavable, thrombin-cleavable and uncleavable (PEG) ACPPs uptake in aorta (a), liver (b) and muscle (c). Y-axis represents the relative fluorescent signal intensity.
