# Supplementary material for: Identification of High-Risk Plaques by MRI and Fluorescence Imaging in a Rabbit Model of Atherothrombosis
Source: PLoS One. 2015 Oct 8;10(10):e0139833. doi: 10.1371/journal.pone.0139833 (PMC4598148; doi:10.1371/journal.pone.0139833)
Supplement: S2 Appendix — (DOCX) [file pone.0139833.s002.docx]

**S2 Appendix: MR-angiogram for planning MRI scans and co-register with fluorescence images and histology.**


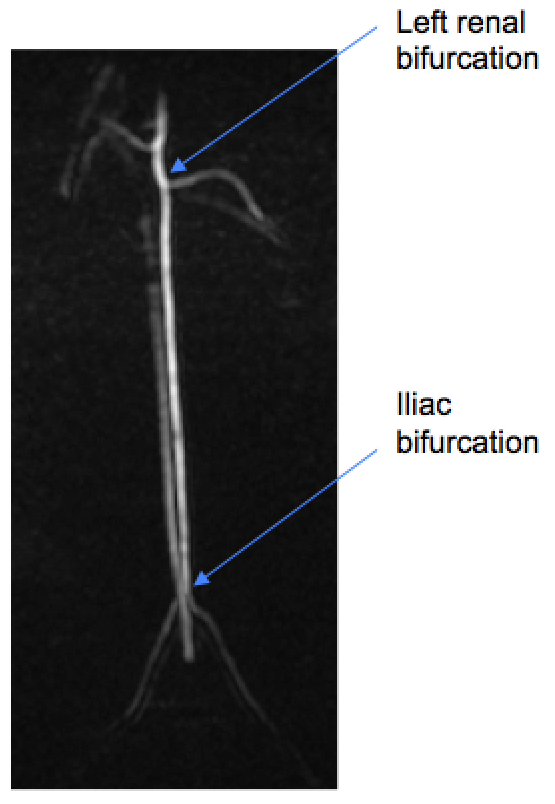
Prior to acquiring T1 Black Blood (T1BB) MRI images, an ungated coronal 3D MR angiogram was acquired as a scout image using a fast field echo (FFE) sequence. The following parameters were used: FOV = 300x150x20 mm^3^, TE = 3.15 ms, TR = 20ms, flip angle = 15°, number of averages = 2, acquisition matrix = 256x244x20. Images were reconstructed using maximum intensity projection (MIP), as shown in the figure below (Fig. S2). The left renal and iliac bifurcations were used as anatomic markers to align pre- and post- MRI scans, fluorescence imaging and histology.

Figure S2: An ungated coronal 3D phase contrast MR angiograms was acquired as scout image for guiding the planning of MRI scans. The left and iliac bifurcations (blue arrows) were used to align scans between pre- and post- triggering MRI, fluorescence scans and histology.
